# Boeravinone B, A Novel Dual Inhibitor of NorA Bacterial Efflux Pump of *Staphylococcus aureus* and Human *P*-Glycoprotein, Reduces the Biofilm Formation and Intracellular Invasion of Bacteria

**DOI:** 10.3389/fmicb.2017.01868

**Published:** 2017-10-04

**Authors:** Samsher Singh, Nitin P. Kalia, Prashant Joshi, Ajay Kumar, Parduman R. Sharma, Ashok Kumar, Sandip B. Bharate, Inshad A. Khan

**Affiliations:** ^1^Clinical Microbiology Division, Indian Institute of Integrative Medicine (CSIR), Jammu, India; ^2^Academy of Scientific and Innovative Research (AcSIR), Indian Institute of Integrative Medicine (CSIR), Jammu, India; ^3^Medicinal Chemistry Division, Indian Institute of Integrative Medicine (CSIR), Jammu, India; ^4^Cancer Pharmacology Division, Indian Institute of Integrative Medicine (CSIR) Jammu, India

**Keywords:** NorA, *P*-glycoprotein, biofilm, *Staphylococcus aureus*, efflux inhibition

## Abstract

This study elucidated the role of boeravinone B, a NorA multidrug efflux pump inhibitor, in biofilm inhibition. The effects of boeravinone B plus ciprofloxacin, a NorA substrate, were evaluated in NorA-overexpressing, wild-type, and knocked-out *Staphylococcus aureus* (SA-1199B, SA-1199, and SA-K1758, respectively). The mechanism of action was confirmed using the ethidium bromide accumulation and efflux assay. The role of boeravinone B as a human *P*-glycoprotein (*P*-gp) inhibitor was examined in the LS-180 (colon cancer) cell line. Moreover, its role in the inhibition of biofilm formation and intracellular invasion of *S. aureus* in macrophages was studied. Boeravinone B reduced the minimum inhibitory concentration (MIC) of ciprofloxacin against *S. aureus* and its methicillin-resistant strains; the effect was stronger in SA-1199B. Furthermore, time–kill kinetics revealed that boeravinone B plus ciprofloxacin, at subinhibitory concentration (0.25 × MIC), is as equipotent as that at the MIC level. This combination also had a reduced mutation prevention concentration. Boeravinone B reduced the efflux of ethidium bromide and increased the accumulation, thus strengthening the role as a NorA inhibitor. Biofilm formation was reduced by four–eightfold of the minimal biofilm inhibitory concentration of ciprofloxacin, effectively preventing bacterial entry into macrophages. Boeravinone B effectively inhibited *P*-gp with half maximal inhibitory concentration (IC_50_) of 64.85 μM. The study concluded that boeravinone B not only inhibits the NorA-mediated efflux of fluoroquinolones but also considerably inhibits the biofilm formation of *S. aureus.* Its *P*-gp inhibition activity demonstrates its potential as a bioavailability and bioefficacy enhancer.

## Introduction

The development of multidrug resistance (MDR) upon chronic exposure to chemotherapeutic agents is considered the major reason for the failure of chemotherapy in cancer and infectious diseases (Poole, 2005; Leitner et al., 2011; Joshi et al., 2014). Efflux pumps are transmembrane proteins, which are the major contributors to multidrug resistance (MDR) in patients with cancer and infectious diseases. *P*-glycoprotein (gp), a multidrug-resistance protein (also called MDR1), accounts for the development of resistance to various drugs as well as for the transport of endogenous and exogenous substrate ligands, such as various toxins, hormones, and drugs (Chen et al., 1986; Joshi et al., 2014). Moreover, *P*-gp alters the pharmacokinetics and pharmacodynamics of its substrates by modulating their absorption, distribution, metabolism, elimination, and toxicity properties (Lin, 2003). Bacterial multidrug efflux pumps are the major contributors to microbial resistance to several classes of antibiotics (Kaatz et al., 2003; Tegos et al., 2011; Kalia et al., 2012).

Presence or augmented expression of efflux pumps is responsible for reduced drug availability to inhibit the specific target. Due to continuous efflux, comparatively lower concentration of antimicrobial agent reaches the target, which may increase the rate of new mutations thus generating newer types resistant mutants exhibiting novel resistance mechanisms (Poole, 2005; Piddock, 2006; Sun et al., 2014). With the continuous emergence of pathogenic resistance to conventional drugs because of efflux through MDR pumps, increasing efforts are directed toward discovering efflux inhibitory molecules. NorA efflux pump is the most widely studied among efflux pumps of *Staphylococcus aureus*. According to a study NorA efflux pump was found to be overexpressed in 43% of resistant strains of *S. aureus* (Patel et al., 2010; Astolfi et al., 2017). NorA, an established multidrug efflux pump model in *S. aureus*, accounts for extruding quinolones, quaternary ammonium salts, acridines, rhodamines, verapamil, and ethidium bromide (Kaatz et al., 2003; Khan et al., 2006; Leitner et al., 2011; Tegos et al., 2011; Holler et al., 2012; Schindler et al., 2013; Fontaine et al., 2015; Tintino et al., 2016). Recently some non-antibiotics derivatives as potent NorA efflux pump inhibitors have been reported utilizing pharmacophore based modeling and drug repurposing utilizing *in silico* approach (Astolfi et al., 2017; Sabatini et al., 2017). In addition, some less toxic benzochromene derivatives and dithiazole thione derivatives have also been reported as NorA efflux inhibitors (Ganesan et al., 2016; Lowrence et al., 2016). A recently published comprehensive review summarized the applications of nanoparticles are also being explored for efflux inhibitory activity in addition to biofilm inhibition (Gupta et al., 2017).

**FIGURE 1:**
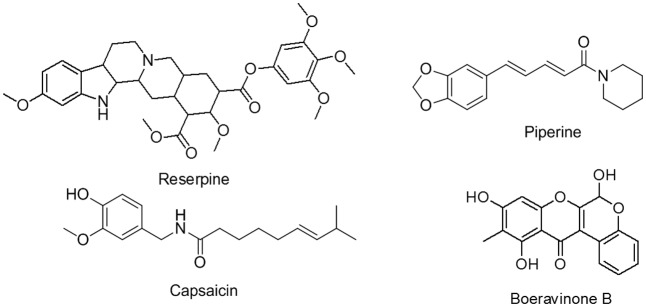
Reserpine, piperine, capsaicin and boeravinone B (molecule used in this study).

Despite their varying structures, the efflux pumps of bacterial and mammalian systems have sufficient substrate homology. Studies have reported many dual types of natural plant products that act as inhibitors, such as verapamil, reserpine, piperine, and capsaicin (**Figure 1**), as well as osthol and curcumin (Bhardwaj et al., 2002; Khan et al., 2006; Kalia et al., 2012; Joshi et al., 2014). These inhibitors offer advantages, such as enhanced gastrointestinal absorption, improved permeation through the blood–brain barrier, increased drug concentrations in mammalian cells for improved killing of invasive pathogens, and the use of lower concentrations of drugs to eliminate side effects (Khan et al., 2006).

In addition to drug efflux, a critical problem is biofilm formation, which poses a major hindrance in the evolution of novel chemotherapeutic agents (Bjarnsholt et al., 2013). Nearly 80% of human bacterial infections are caused by biofilm formation, and 60% of nosocomial infections are caused by biofilm formation on medical devices and implants (Darouiche, 2004; Hou et al., 2012). Biofilm eradication requires a much higher concentration, namely 10–1000 times the minimum inhibitory concentration (MIC), of antibiotics. Active efflux has been reported to contribute to increased resistance in bacterial biofilms (Lewis, 2001; Kvist et al., 2008; Baugh et al., 2014; Van Acker and Coenye, 2016; Sabatini et al., 2017). Biofilms have slow metabolism and growth, the main reasons for increased resistance, because most antibiotics target metabolic or cell division pathways (Lewis, 2007, 2008). Researchers have reported that the synergistic action of molecules abolishes biofilm formation (Baugh et al., 2012).

*Staphylococcus aureus* is considered an extracellular pathogen, but there are accumulating evidences to reveal that *S. aureus* causes severe intracellular infections, such as endocarditis and septic shock (Hooper, 2005). This organism, on getting an opportunity to invade, survives and promotes diseases. Hirakata et al. (2009) and Kalia et al. (2012) concluded that efflux pump-overexpressing strains can easily invade cells.

Several natural plant products act as efflux pump inhibitors (Stavri et al., 2007). Boeravinone B, the compound of interest in the present study, was isolated from the roots of *Boerhavia diffusa*, a plant belonging to the Nyctaginaceae family (Patil and Bhalsing, 2016). This plant is also known as Punarnava in the Indian traditional system of medicine, Ayurveda, and is established for its medicinal properties. In ancient times, plants were used to cure various physiological conditions, such as inflammation, jaundice, dyspepsia, nephrotic syndrome, convulsions, spleen enlargement, abdominal pain and stress, nematodal and microbial infections, and asthma (Ahmed-Belkacem et al., 2007; Khan et al., 2013; Bairwa et al., 2014). Boeravinone C and G have been reported to possess *P*-gp inhibitory activity (Ahmed-Belkacem et al., 2007; Stacy et al., 2013).

In this study, we described the role of boeravinone B in ciprofloxacin potentiating activity against NorA-overexpressing strains of *S. aureus* and *P*-gp inhibition in the colon cancer cell line.

## Materials and Methods

### Chemicals

All fluoroquinolones, ethidium bromide reserpine, piperine, fluorescein isothiocyanate (FITC) and 4′,6-diamidino- 2-phenylindole (DAPI) were purchased from Sigma Chemical Co. (St. Louis, MO, United States). Microbiological media were purchased BD Microbiology Products (Sparks, MD, United States). All other chemicals were of analytical grade.

### Bacterial Strains

*Staphylococcus aureus* ATCC 29213, SA-1199B, SA-1199, SA-K1758, MT23142, and MRSA15187 were cultivated on trypticase soya agar (TSA). Important characteristics are described in **Table 1**.

**Table 1:** Characteristics of *Staphylococcus aureus* strains used in this study.

Strain code	Characteristic	Origin
SA-1199	Wild type	Clinical isolate (endocarditis)
SA-1199B	NorA overexpressing	Ciprofloxacin resistant derivative of SA-1199
SA-K1758	NorA knockout	Derivative of NCTC-8325-4
MT23142	NorA overexpressing	Derivative of NCTC-8325
MRSA15187	MRSA	MRSA
ATCC 29213	MSSA	Standard strain; methicillin sensitive

### Boeravinone B Isolation

Authentic samples of *B. diffusa* roots were collected from Jammu region (Voucher Specimen No. 21713), dried and powdered followed by solvent extraction in dichloromethane: methanol mixture (1:1 v/v) using cold maceration method. Crude extract was concentrated and purified by repeated silica gel column chromatography using hexane: ethyl acetate (95: 5 to 75: 25 v/v).

### Drug Potentiation by Broth Checkerboard Microdilution

Drug potentiation or synergistic action of compound was assessed by broth checkerboard microdilution technique, which is most known method (Eliopoulos and Wennersten, 2002). Combination of ciprofloxacin and boeravinone B was tested in Mueller Hinton Broth (MHB; pH 7.0) against all bacterial strains using 96 well microtitre plates at a concentration range of 0.06–32 μg/ml and 0.8–50 μM, respectively. Additionally, combination of ethidium bromide and boeravinone B was assessed in similar way using *S. aureus* SA-1199B. Cell suspension of a density of 0.5 McFarland (≈1.5 × 10^8^ cfu per ml of *Escherichia coli*) was used as inoculum after diluting 1:100, a volume of 0.1 ml (5 × 10^5^) was added to plates. The plates were incubated at 37°C for 18–20 h. Reserpine and piperine (known efflux pump blocker) were used as the control in this study. Minimum effective concentration (MEC) of boeravinone B was determined which is defined as concentration at which there is atleast fourfold reductions in MIC of ciprofloxacin. In a similar way various other fluoroquinolones and ethidium bromide were also assessed for synergistic potential of boeravinone B.

### Time Kill Curve

Time-kill study of ciprofloxacin alone and in combination with boeravinone B was performed in 50 ml volume conical flasks containing 25 ml of MHB using the previously described method (Eliopoulos and Moellering, 1996). Ciprofloxacin at 2 μg/ml (0.25 × MIC) was tested alone and in combination with boeravinone B at the MEC concentration (12.5 μM) as determined above. Ciprofloxacin was also tested alone at an MIC of 8 μg/ml. NorA overexpressing *S. aureus* SA-1199B was used as the test bacterium. Ten folds serial dilutions of these aliquots were spotted on TSA plates.

### Impact on Frequency of Resistance

Frequency of resistance (FOR) or Mutation prevention concentration (MPC) of ciprofloxacin against *S. aureus* ATCC 29213 was determined by method described elsewhere (Drugeon et al., 1999). MHA plates were supplemented with MIC, 2×, 4×, and 8× MIC of ciprofloxacin’s concentrations with or without boeravinone B at MEC. A bacterial suspension of 0.1 mL (10^9^ cfu) was plated on to each ciprofloxacin containing plates without and with boeravinone B. MPC was determined as the ratio of colonies appearing on ciprofloxacin or ciprofloxacin with boeravinone B combination to total colonies plated on a drug free plate.

### Ethidium Bromide Efflux and Accumulation Studies

Fluorescence based method as described by Brenwald et al. (1998) was used to determine the ethidium bromide efflux inhibitory as well accumulation studies. For this purpose a freshly grown NorA overexpressing *S. aureus* SA-1199B was suspended to an optical density of 0.2 in uptake buffer (110 mM NaCl, 7 mM KCl, 50 mM NH_4_Cl, 0.4 mM Na_2_HPO_4_, 52 mM Tris base and 0.2% glucose, adjusted to pH 7.5 with HCl). Cells were loaded with 10 μg/ml ethidium bromide for 30 min at 37°C. Extracellular ethidium bromide was removed by centrifugation and pellet was resuspended in same volume of uptake buffer with and without boeravinone B (12.5 μM). Reserpine at 25 μg/ml was used as a known efflux pump inhibitor. For accumulation studies bacterial cells loaded with ethidium bromide for 30 min after which EPIs (boeravinone B and reserpine) were added. Loss or gain of fluorescence in presence of compound and known EPI was deduced at 530 and 600 nm for excitation and emission wavelength, respectively, using multimode reader Infinite 200 Pro (Tecan Mannedorf, Switzerland).

### Biofilm Inhibition

Effect of boeravinone B on the biofilm inhibitory potential of ciprofloxacin was studied with drug combination on biofilm formation, as previously described (Schillaci et al., 2010). For biofilm formation, cultures (*S. aureus* SA-1199 and SA-1199B) were grown overnight using TSB supplemented with 2% glucose (TSBG) and diluted to an optical density (570 nm) of 0.015 after diluting 1:200 (1 × 10^9^ cells/mL). Microtitre plates for inhibitory studies were prepared as described for synergistic activity with ciprofloxacin by checkerboard microdilution method. After 24 h, wells were washed thrice with PBS followed by fixation with methanol for 15 min and finally air-dried in inverted position at 37°C. The wells of the dried plates were stained with 0.1% (w/v) crystal violet for 10 min and rinsed thoroughly with water until the negative control wells (without biofilms) appeared colorless. To quantify biofilm formation, 0.2 mL of 95% ethanol was added to wells of plate that were stained with crystal violet. Immediately absorbance read at 595 nm using microplate reader. In a separate plate the contents were decanted and washed with PBS to remove the planktonic cells and again filled with fresh TSB. After incubation for 30 min, 15 μl of 0.04% resazurin and 12.5 μl of 20% Tween80 were added in each well of the plate to access the viability of the cells within biofilm. The plates were incubated for 15 min and fluorescence was determined at an excitation at 560 nm and emission at 590 nm (Tecan Infinite M200 microplate reader) as described previously (Bauer et al., 2013). Minimal biofilm inhibitory concentration (MBIC) is defined as the concentration at which absorbance was reduced by ≥90% or was nearly equivalent to media control wells.

### Confocal Microscopic Studies for Biofilm Inhibition

The potentiating effect of boeravinone B was further confirmed by staining the biofilms with FITC and DAPI which stains protein (amine reactive) and nucleic acid (DNA), respectively, by modified method as described by Yang (using DAPI in place of Hoechst) followed by visualization by confocal laser scanning microscopy (Yang et al., 2015). The biofilm of *S. aureus* ATCC 29213 was grown in six well poly styrene plate (Nunc) containing sterile 18 mm glass cover slips. Ciprofloxacin was used at 4 μg/ml which is MBIC and at 1 μg/ml which is subMBIC (0.25× MBIC) as determined above. Boeravinone B at 12.5 μM (MBIC) was added to sub-inhibitory concentration of ciprofloxacin (0.25× MBIC). The plates were incubated for 24 h at 37°C. PBS washes were given to eliminate planktonic cells followed by fixation of biofilm with 5% para-formaldehyde for 1 h at 50°C. Fixed biofilm was flooded with 0.001% (w/v) FITC and 1 μg/ml DAPI at kept at room temperature for 1 h. Images were seen and captured on 40× oil immersion lens using CLSM (Olympus flou View 1000).

### *In Vitro* Screening of *P*-Glycoprotein Inhibitory Activity

*P*-glycoprotein inhibitory activity was assessed by the method described earlier using colorectal cell line LS180 (Jin et al., 2012; Joshi et al., 2014). Cell monolayer was prepared in 96 well plate using Dulbecco’s Modified Eagle’s medium (DMEM) media. The media was decanted and replaced with Hank’s buffer containing 10 mM of Rh123 as a *P*-gp substrate. Twofold serial dilutions of boeravinone B (80–5 μM) were added and plate was incubated at 37°C in CO_2_ incubator for 90 min. A known *P*-gp inhibitor elacridar (10 μM) was used as positive control. The contents of the wells were decanted and washed four times with cold PBS followed by cell lysis for 1 h using 200 μl of lysis buffer (0.1% Triton X 100 and 0.2 N NaOH). A total of 100 μl of lysate was used for reading fluorescence of Rh123 at an excitation at 485 nm and emission at 590 nm. All samples were normalized by dividing fluorescence of each sample with total protein present in the lysate. Half minimal inhibitory concentration (IC_50_) value of boeravinone B was calculated using Graphpad Prism 5 software (GraphPad Software, Inc., La Jolla, CA, United States). Data is expressed as mean ± SD or representative of one of three similar experiments. Comparisons were made between control and treated groups or the entire intra group using one way ANOVA with post Bonferroni test through GraphPad Prism 5.00.288 statistical analysis software. ^∗^*p*-values <0.05 were considered significant.

### Molecular Docking Studies against *P*-Glycoprotein and NorA Efflux Pumps

Due to unavailability of crystal structures of human *P*-gp and *S. aureus* NorA, molecular docking studies were carried out using the homology models. The homology models for human *P*-gp (Jin et al., 2012) was prepared from *Caenorhabditis elegans P*-gp (PDB ID: 4AZF) and *S. aureus* NorA efflux pump (Aller et al., 2009) was prepared from glycerol 3-phosphate transporter pump from *E. coli* (PDB ID: IPW4) using Glide Induced Fit Docking in default setting. For Nor A docking studies, ciprofloxacin/reserpine binding site was used to construct grid file whereas for *P*-gp docking, verapamil binding site was used (Aller et al., 2009).

### Macrophage Invasion Assay

To assess the impact of boeravinone B on invasion of *S. aureus* in macrophage, previously described method was used (Cheung and Bayles, 2007). Murine macrophage cell line J774 maintained on Roswell Park Memorial Institute medium (RPMI) supplemented with 10% (v/v) fetal calf serum was used as host. The macrophages were grown in monolayer to a density to 10^5^ cells/well in 24 well plate in physiological conditions, i.e., 37°C and 5% CO_2_ for 24 h. 10^6^ cfu/well of overnight grown *S. aureus* strains SA-1199B, SA-1199, and SA-K1758 were used to infect macrophages (MOI of 10) in presence and absence of boeravinone B at its MEC (12.5 μM) for 2 h. Extracellular and adherent bacteria washed with PBS (pH 7.4) followed by treatment with 50 μg/ml gentamicin for 30 min. Intracellular bacteria were quantified by host cell lysis by brief treatment of 0.07% SDS which followed neutralization with 6% BSA. Viable bacteria were counted by plating on TSA. In a similar experiment RPMI devoid of FCS was used to assess the role of FCS.

## Results

### Isolation and Purity of Boeravinone B

By using the cold maceration method, 6,9,11-trihydroxy-10-methylchromeno[3,4-b]chromen-12(6H)-one (boeravinone B; **Figure 1**) was isolated from the roots of *B. diffusa*. It is a yellow solid compound, with a melting point of 201°C–203°C. The structure was confirmed through high-resolution mass spectroscopy (m/z 313.0708 [M + H^+^], as calculated for C_17_H_12_O_6_ + H^+^: 313.0707) and comparison of the ^1^H and ^13^C NMR data. Boeravinone B used in this study had 96% purity, as determined through high performance liquid chromatography. Data related to the purity and structure of the isolated boeravinone B was characterized by comparison of spectral data with described reported values (Kadota et al., 1989) (Supplementary Data S1).

### Effect of NorA Efflux Pump Inhibition on MIC of Fluoroquinolones and Ethidium Bromide

The resistance reversal activity of boeravinone B was quantified by calculating the fold reduction in the MIC of ciprofloxacin. Boeravinone B lack anti-staphylococcal activity when assessed up to 100 μM against all tested strains. This MIC of ciprofloxacin alone and in combination with boeravinone B was determined (**Table 2**). Boeravinone B reduced the MIC of ciprofloxacin by twofold in *S. aureus* SA-1199 and ATCC 29213, whereas the effect was stronger in the NorA-overexpressing *S. aureus* strains SA-1199B and MT-23142, where the MIC was reduced by eightfold. Conversely, SA-K1758 (NorA knocked out) did not show any reduction in the MIC of ciprofloxacin. Boeravinone B was also active against a methicillin-resistant *S. aureus* (MRSA) strain, which showed a fourfold reduction in the ciprofloxacin MIC.

**Table 2:** Ciprofloxacin potentiation by boeravinone B against various strains of *S. aureus* with different levels of NorA expressions and MRSA.

Compounds	EPI^#^ μg/ml (μM)	MIC (μg/ml) of ciprofloxacin					
		
		SA1199	SA-1199B	SA-K 1758	ATCC 29213	MT-23142	MRSA-15187
Ciprofloxacin	0	0.25	8	0.12	0.25	0.25–0.5	32
Boeravinone B	8 (25)	0.12	1	0.12	0.12	0.03	8
Piperine	50 (88)	0.12	4	0.12	0.06	ND	16
Reserpine	25 (41)	0.06	2	0.12	0.12	ND	ND


*^#^All EPIs tested have no self-antimicrobial activity up to 100 μM.*

In addition to ciprofloxacin, spectrum of activity was also assessed using other fluoroquinolones like ofloxacin and norfloxacin which are well known substrates for NorA efflux pump and newer fluoroquinolones like gatifloxacin and moxifloxacin which are poor efflux substrates for NorA. Boeravinone B was used at 4× MEC for ethidium bromide (6.25 μM or 2 μg/ml). Potential of boeravinone B to inhibit the efflux of other fluoroquinolones was investigated using *S. aureus* strains SA-1199B, SA-1199, SA-K1758, and MT23142 (**Table 3**). MIC potentiation using ethidium bromide yielded similar effects of eightfold reduction in the MIC of ethidium bromide (2 μg/mL) compared with that of ethidium bromide alone, which exhibited an MIC of 16 μg/mL against SA-1199B. In NorA overexpressing strains (SA-1199B and MT23142), boeravinone B reduced the MIC of norfloxacin by eightfold while only twofold reduction in MIC of ofloxacin, moxifloxacin, and gatifloxacin was observed. It was observed that ciprofloxacin MIC reduction was relatively less (two–fourfold) in case of wild type *S. aureus* SA-1199 against these fluoroquinolones. There was no synergistic effect against *S. aureus* SA-K1758 due to absence of NorA efflux pump. Significant reduction in MICs against NorA overexpressing strains of *S. aureus* concludes that ciprofloxacin and norfloxacin have stronger affinity for NorA and efflux can be inhibited using boeravinone B.

**Table 3:** Activity of boeravinone B on the MICs of ethidium bromide and other fluoroquinolones in susceptible and resistant *S. aureus* strains.

Antibacterial agents	Boeravinone B concentration in μg/ml (μM)	MIC (μg/ml) for given *S. aureus* strain			
		
		SA-1199B	SA-1199	SA-K1758	MT23142
EtBr	0	32	1	0.5	2
	8 (25)	2	0.25	0.12	0.12
Norfloxacin	0	32	0.25	0.12	32
	8 (25)	4	0.12	0.12	4
Ofloxacin	0	1	1	1	0.5
	8 (25)	0.5	1	0.5	0.5
Moxifloxacin	0	0.25	0.06	0.12	0.06
	8 (25)	0.12	0.06	0.12	0.06
Gatifloxacin	0	1	1	0.12	0.06
	8 (25)	0.25	0.5	0.12	0.06


### Effect of Boeravinone B on Time–Kill Kinetics of Ciprofloxacin

**FIGURE 2:**
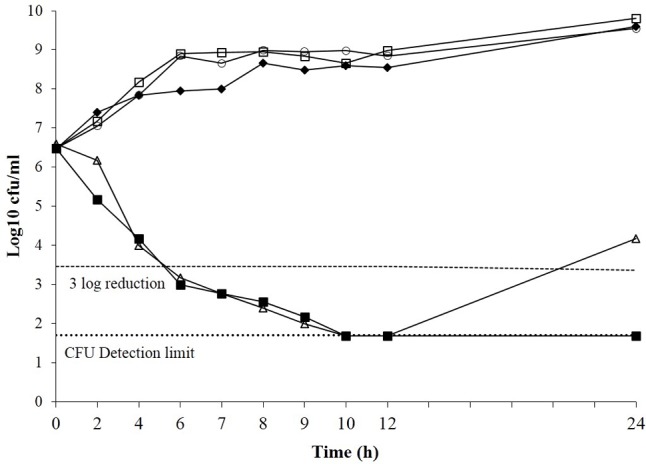
Time kill curve of *Staphylococcus aureus* SA-1199B showing the bactericidal effect of ciprofloxacin alone (open triangle), subMIC alone (closed diamond) and ciprofloxacin at subMIC in combination with boeravinone B at MEC (closed square) including boeravinone B alone (Open circle). Growth control having plain media without ciprofloxacin or EPI served control (Open square). Each time point is mean Log_10_ of three independent readings.

The effect of boeravinone B on the time–kill kinetics of ciprofloxacin was assessed in *S. aureus* SA-1199B. Ciprofloxacin alone (MIC: 8 μg/mL) showed 3-log reduction in growth, whereas the same was accomplished at a subinhibitory concentration of the ciprofloxacin (0.25 × MIC) in combination with boeravinone B at a minimum effective concentration (MEC; 12.5 μM). The sub MIC of ciprofloxacin alone could not reduce the growth. Ciprofloxacin alone at an MIC effectively reduced the colony-forming units (CFU) in 6 h, but cell growth reoccurred after 24 h. The combination of ciprofloxacin (at subinhibitory concentration) and boeravinone B prevented the growth of resistant colonies (**Figure 2**).

### Frequency of Ciprofloxacin Resistance in Presence of Boeravinone B

For effective clinical applications, drugs should be able to suppress mutations and resistance mechanisms. A resistant mutant selection study was performed on wild-type *S. aureus* ATCC 29213 because this strain does not have any known mutations in the regulatory region of NorA and drug targets (DNA gyrase and topoisomerase IV). The minimum concentration of a drug at which no resistant mutant is selected is defined as its MPC. Ciprofloxacin alone showed an MPC of 4 μg/ml whereas, in combination with 12.5 and 25 μg/ml of boeravinone B, the MPC of ciprofloxacin reduced to 2 and 1 μg/ml, respectively (**Table 4**). The MPC of this combination was lower than the *C*_max_ of ciprofloxacin (3–4 μg/ml) in human plasma, indicating the clinical relevance of these combinations in restricting the selection of resistant mutants.

**Table 4:** Mutation prevention concentration (MPC) of ciprofloxacin with boeravinone B against *S. aureus* ATCC 29213.

Boeravinone	Mutation frequency with ciprofloxacin			
	
B conc. (μM)	2 × MIC	4 × MIC	8 × MIC	16 × MIC
0	5 × 10^-8^	8 × 10^-9^	4 × 10^-9^	<10^-9^
12.5	2.2 × 10^-9^	<10^-9^	<10^-9^	<10^-9^
25.0	<10^-9^	<10^-9^	<10^-9^	<10^-9^


### Effect of Boeravinone B on Ethidium Bromide Efflux Inhibition and Accumulation

**FIGURE 3:**
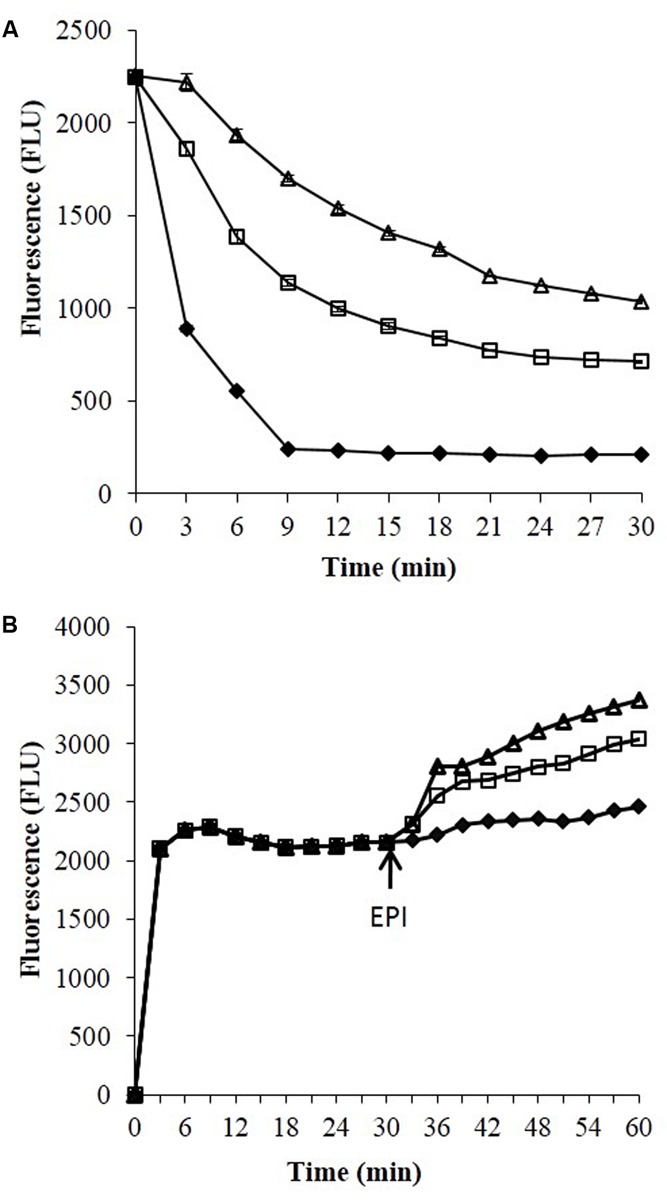
Ethidium bromide studies efflux **(A)** and accumulation **(B)** studies against *S. aureus* SA-1199B. Symbols represents control which have no addition of any EPI (closed diamond), in presence of boeravinone B (open square) and in presence of known EPI, i.e., reserpine (open triangle).

**FIGURE 4:**
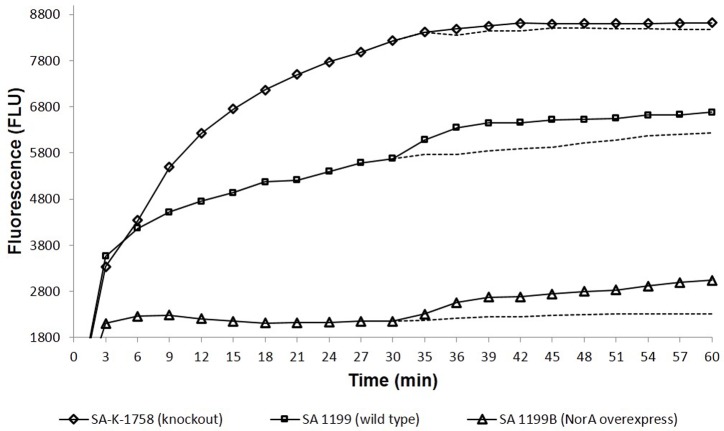
Comparative accumulation of EtBr in presence of Boeravinone B against *S. aureus* SA-1199B (triangle), SA-1199 (square) and SA-K1758 (diamond) having different levels of NorA expressions, i.e., overexpression, wild type and knockout, respectively. Dotted lines after 30 min indicates the respective controls in absence of boeravinone B.

Ethidium bromide is a well-known substrate for the NorA MDR efflux pump. SA-1199B cells were allowed to be loaded with ethidium bromide with and without boeravinone B and placed in a fluorometer cuvette containing fresh medium. Ethidium bromide fluoresces only when bound to nucleic acids in cells; therefore, a rapid decrease occurred in the fluorescence because of the NorA-mediated efflux of ethidium bromide. As shown in **Figure 3**, only the control cells without boeravinone B maximally extruded ethidium bromide, resulting in significantly decreased fluorescence in the assay period. Conversely, in cells treated with boeravinone B, the efflux of ethidium bromide was prevented, resulting in the prolonged retention of fluorescence. In the accumulation studies, ethidium bromide uptake was allowed in bacterial cells for 30 min. A counterbalance effect occurred because of the simultaneous efflux of ethidium bromide, which effectively increased the fluorescence. The addition of boeravinone B at this stage resulted in a surge in the fluorescence because of the inhibition of efflux pumps, resulting in a higher retention of fluorescence (**Figure 3**). Reserpine, a known efflux pump inhibitor, was used as the positive control in both studies. When compared, ethidium bromide accumulation in SA-1199B, SA-1199, and SA-K1758, accumulation was maximum in knockout followed by wild type and overexpressing which is in correlation with the phenotypes of the tested strains. In presence of boeravinone B, accumulation was significantly increased in NorA overexpressing, while minor effect was seen for wild type. *S. aureus* SA-1199. There was no effect on the accumulation efficiency of SA-K1758 which is devoid of functional *norA* gene (**Figure 4**).

### Biofilm Inhibition

The minimum concentration of a drug for inhibiting a biofilm is defined and expressed as its MBIC. The MBIC of a drug is typically higher than its MIC because of the lesser permeability of the drug within the matrix of a biofilm. In this study, ciprofloxacin showed an MBIC of 4 and 16 μg/mL for *S. aureus* SA-1199 and SA-1199B, respectively. A concentration-dependent decrease occurred in the MBIC of ciprofloxacin against both strains in the presence of boeravinone B (6.25–25 μM; **Table 5**). To ascertain that the biofilm inhibition was caused by the killing of the bacteria embedded in the matrix of biofilm, the resazurin staining of a parallel set of plates was performed. The change in the color of this redox dye from blue to red indicated the viability of the cells in the well. The biofilm inhibition correlated well with the cell viability (Supplementary Figure S1).

**Table 5:** Effect on biofilm inhibitory potential of ciprofloxacin when combined with boeravinone B at different concentrations against various strains of *S. aureus*.

Boeravinone B (μM)	MBIC (μg/ml) of ciprofloxacin		
	
	SA-1199	SA1199B	*S. aureus* ATCC 29213
0	4	16	4
6.25	2	8	2
12.5	1	4	1
25	1	2	1


### Microscopic Studies

**FIGURE 5:**
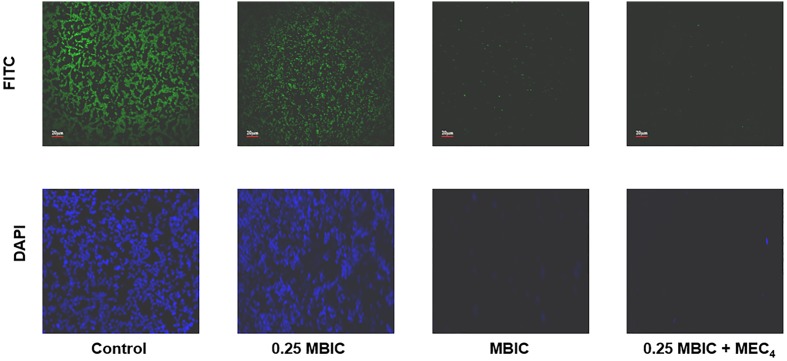
CLSM of biofilm; untreated, treated with ciprofloxacin at subMBIC (0.25 × MBIC) and MBIC alone. Last column shows the presence of boeravinone B combined with subMBIC of ciprofloxacin. Upper line is stained with FITC and lower with DAPI.

Effect of boeravinone B plus ciprofloxacin was evaluated by microscopic studies of the *S. aureus* ATCC 29213-generated biofilm. Fluorescein isothiocyanate stains proteins and imparts green fluorescence, whereas 4′,6-diamidino-2-phenylindole binds to nucleic acids and yields blue fluorescence when observed in the whole image field. As shown in **Figure 5**, the untreated biofilms were associated with a large amount of proteins and DNA. The sub MBIC of ciprofloxacin did not inhibit the biofilms; however, in combination with the MEC of boeravinone B, ciprofloxacin inhibited the biofilm, as did the MBIC of ciprofloxacin, as evident by the reduced intensity of green and blue fluorescence.

### Molecular Docking on *P*-gp

**FIGURE 6:**
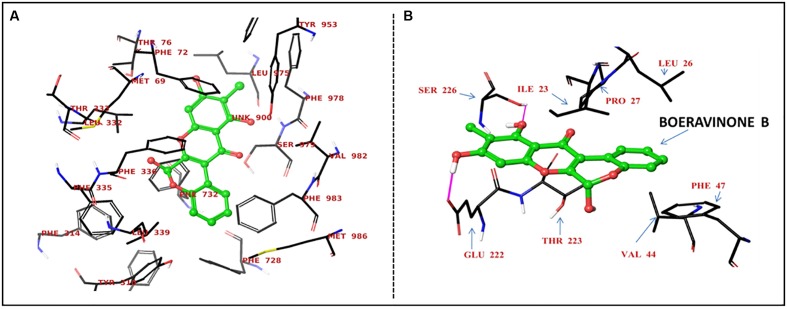
Proposed binding site of boeravinone B with human *P*-gp **(A)** and *S. aureus* NorA efflux pumps **(B)**.

Boeravinone B was observed to interact with *P*-gp at the verapamil binding site, particularly with the residues Met69, Phe72, Tyr307, Tyr310, Phe314, Leu332, Phe335, Phe336, Leu339, Phe728, Phe759, Phe957, Phe978, and Val982 through hydrophobic π–π interactions (**Figure 6A**). Consequently, it led to an increase in the intracellular accumulation of the substrate Rh123. Similarly, the NorA efflux pump is a transmembrane pump, in which ciprofloxacin or reserpine optimally bind to site 1 through strong hydrogen bonding of carboxyl functionality with the Arg98 cationic guanido group. The hydrophobic and hydrogen interactions of ciprofloxacin were mimicked by boeravinone B. Molecular modeling studies with the bacterial NorA efflux pump homology model revealed that boeravinone B binds to the Ile23 and Glu222 residues of the pump through strong hydrogen bonding formed by phenolic hydroxyl groups and thus block the interaction of substrate quinolones with the efflux binding cavity (**Figure 6B**).

### Invasion Inhibition and Role of Fetal Calf Serum

**FIGURE 7:**
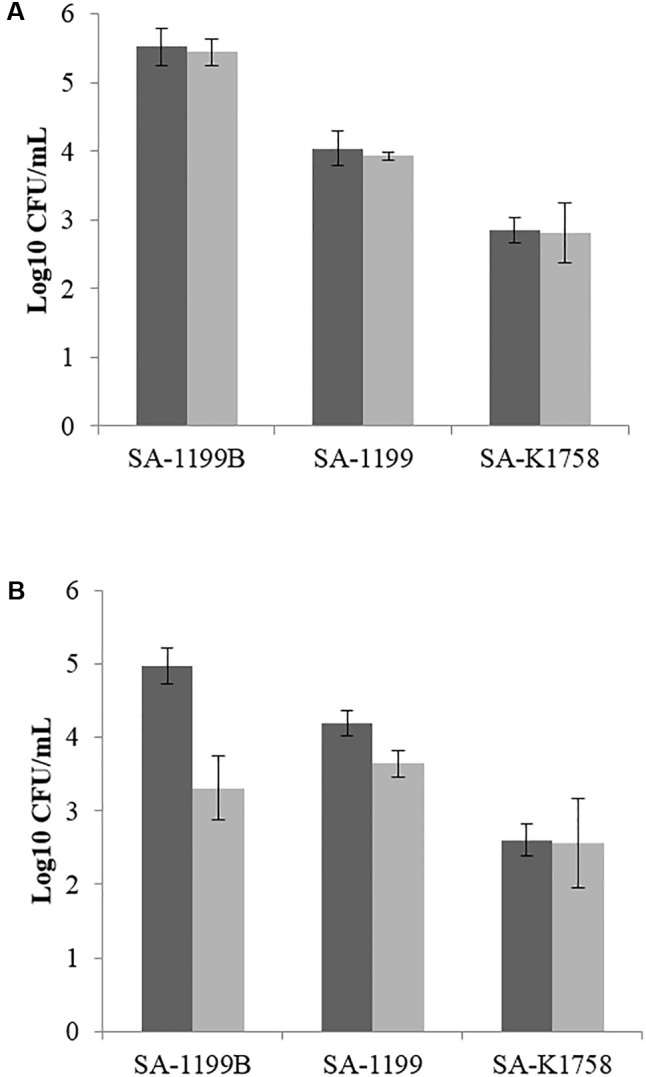
Macrophage invasiveness by various strains of *S. aureus* in presence (gray bars) and absence (black bars) of boeravinone B. Assay was performed using standard RPMI media with **(A)** and without FCS **(B)**.

The intracellular invasion of *S. aureus* SA-1199B was nearly 1.5 log_10_ higher than that of *S. aureus* SA-1199 in the murine macrophage cell line J774. Boeravinone B at its MEC (12.5 μM) was not active when the experiment was performed in the presence of 10% fetal calf serum (FCS). When the experiment was repeated using RPMI media without FCS, the invasiveness of *S. aureus* had reduced, suggesting the binding of boeravinone B to FCS. The invasiveness of SA-1199B showed greater reduction (1.6 log_10_) than did that of *S. aureus* SA-1199 (0.5 log_10_). *S. aureus* SA-K1758 exhibited the least penetration into macrophages, and boeravinone B did not affect the invasiveness of this strain (**Figure 7**).

## Discussion

Efflux pumps are membrane-bound proteinaceous transporters known for extruding chemotherapeutic agents or xenobiotics, which are otherwise harmful for bacterial survival. During evolution, microbes were selected and evolved through antibiotic pressure with the overexpression of efflux pumps, which renders the antibiotics inefficient and hence results in the poor efficacy of chemotherapeutic agents. Efflux pumps can export a large pool of structurally unrelated substrates out of cells, which hampers their clinical efficacy. NorA is an MDR efflux pump of the major facilitator superfamily pumps and has 12 transmembrane segments. It is established for expelling various fluoroquinolones. NorA has been well established as a model system for studying efflux pump inhibition in Gram-positive pathogens (Kaatz, 2005; Kumar et al., 2008).

In the present study, boeravinone B obtained from *B. diffusa* was assessed for its NorA inhibitory activity in SA-1199B, which is a model organism for NorA inhibition studies because it mimics the resistance induced by fluoroquinolone pressure. Ethidium bromide MIC potentiation by 16-fold in NorA overexpressing strains in presence of boeravinone B shows inhibition of efflux mediated resistance. Presence of boeravinone B also exhibited two–eightfold reduction in MIC of ciprofloxacin and norfloxacin in these strains. The potentiating effect was less prominent (twofold) in wild type strain *S. aureus* SA-1199. Additionally there was no reduction in MIC of *S. aureus* SA-K1758 which is lacking functional *norA* gene. This concludes that ciprofloxacin and norfloxacin fluoroquinolone are favorable substrate for NorA. Similar observation about the enhanced affinity of NorA for these fluoroquinolones have been reported previously (Gibbons et al., 2003; Mullin et al., 2004). Boeravinone B increased the intrinsic susceptibility of *S. aureus* to ciprofloxacin and significantly reduced the emergence of ciprofloxacin-resistant *S. aureus*. A subinhibitory concentration of ciprofloxacin (2 μg/ml) with the MEC of boeravinone B (12.5 μM) exhibited a bactericidal effect and a 3-log_10_ reduction in CFU in 6 h, with no emergence of mutants even after 24 h. Conversely, ciprofloxacin alone exhibited the same effect at 8 μg/mL, but resistant mutants were observed after 24 h (**Figure 2**). Ethidium bromide efflux inhibition and accumulation are well-known mechanism-based descriptors for the specificity of NorA inhibition. The fluorescence-based efflux accumulation studies of ethidium bromide-preloaded NorA-overproducing *S. aureus* cells revealed reduced efflux in the presence of boeravinone B, indicating the inhibition of the ethidium bromide efflux mechanism. Furthermore, its effect on the retention of Rh123 in *P*-gp-overexpressing human adenocarcinoma LS-180 cells confirmed its role as a dual inhibitor of *P*-gp and the *S. aureus* efflux pump inhibitor. Studies have reported several similar dual inhibitors, such as osthol, curcumin, piperine, and capsaicin (Khan et al., 2006; Kalia et al., 2012). Docking results with the NorA homologous structure and *P*-gp obtained using Glide clearly elaborated that boeravinone B interacts with both pumps through core hydrophobic π–π interactions and hydrogen bonding.

Both methicillin susceptible *S. aureus* and MRSA are common pathogenic concerns in the hospital environment (Livermore et al., 2015). MRSA remains a major concern in the clinical treatment of *S. aureus*-related infections since half a century. Vancomycin was the only drug of choice for treating the increased incidence of MRSA, but resistance abolished the drug’s mechanism of action. With time, novel candidates were observed to be active against these strains, such as daptomycin, linezolid, and some glycopeptides, but resistance to these drugs was reported (Nannini et al., 2010). Because of frequent reports on resistance to antimicrobials, researchers are now using adjuvant therapy as a treatment strategy (Drugeon et al., 1999).

Active efflux has been reported to be associated with increased resistance in bacterial biofilms (Lewis, 2007; Van Acker and Coenye, 2016). The treatment of biofilm-related infections is a major unmet clinical concern because currently used antibiotics are insufficient to treat these highly persistent infections; some candidate molecules are under early development. Evidently, biofilms have slow metabolism and cell growth, which contribute to increased resistance because most currently used antibiotics have been discovered by targeting metabolic or cell division pathways. The synergistic action of molecules was reported to abolish the formation of biofilms (Baugh et al., 2014). These ideas prompted the assessment of the role of boeravinone B in the prevention and eradication of biofilms, a major clinical problem in the current era of anti-infective drug discovery (Bharate et al., 2015). Current studies have revealed that in combination with boeravinone B, the sub MBIC of ciprofloxacin inhibited the formation of biofilms through enhanced drug accumulation. Therefore, boeravinone B could penetrate the complex matrix of *S. aureus*-generated biofilms to inhibit viable bacteria, as observed in the resazurin assay (Supplementary Figure S1). The involvement of efflux pump inhibitors, such as certain diamines, was reported in reducing the invasiveness of *Pseudomonas aeruginosa* in eukaryotic cells (Hirakata et al., 2009). We obtained the similar results for capsaicin in a previous study (Kalia et al., 2012). This observation shows similar effects of boeravinone B, as expected, after the removal of FCS from the assay media (RPMI).

## Conclusion

Boeravinone B is a novel dual inhibitor of bacterial NorA efflux pump and human *P*-gp. Such inhibitors play potential role in drug combinations by retaining higher concentrations of drugs in bacterial and host cells.

## Author Contributions

SS and NK performed microbiological studies. PJ performed isolation of compound and *in silico* studies. AsK and PS performed microscopic studies. SS and IK participated in design and execution of microbiological studies. SB participated in isolation and identification of compound used in this study. PJ and AjK participated in *P*-glycoprotein efflux inhibition studies. IK and SS prepared the manuscript and all authors participated in revising manuscript. All authors approved the study. All authors are accountable for all aspects of work in ensuring that questions related to the accuracy or integrity of the part of work are appropriately investigated and resolved.

## Conflict of Interest Statement

The authors declare that the research was conducted in the absence of any commercial or financial relationships that could be construed as a potential conflict of interest.
