# Supplementary material for: Boeravinone B, A Novel Dual Inhibitor of NorA Bacterial Efflux Pump of Staphylococcus aureus and Human P-Glycoprotein, Reduces the Biofilm Formation and Intracellular Invasion of Bacteria
Source: Front Microbiol. 2017 Oct 4;8:1868. doi: 10.3389/fmicb.2017.01868 (PMC5632727; doi:10.3389/fmicb.2017.01868)
Supplement: Supplementary file 1 [file Data_Sheet_1.doc]

**Boeravinone B, a novel dual inhibitor of NorA bacterial efflux pump of *Staphylococcus aureus* and human P-glycoprotein, reduces the intracellular invasion of bacteria**

Samsher Singh^a,d^, Nitin Pal Kalia^a,#^, Prashant Joshi^b,d^, Ajay Kumar^c^, Parduman R. Sharma^c,d^, Ashok Kumar^c,d^, Sandip B. Bharate^b,d^, Inshad Ali Khan^a,d^,*

**Supporting information**

**Data S1: Data related to purity and structure of Boeravinone B.**

**Brief overview of identification and characterization of boeravinone B**

The structure of isolated compound was characterized by comparison of spectral data with literature values (1). *6,9,11-Trihydroxy-10-methylchromeno[3,4-b]chromen-12(6H)-one (boeravinone B):* yellow solid; m.p. 201-203 °C; HPLC purity: 96% (*t*_R_ = 13.46 min); yield: 3.2 g; IR (CHCl_3_): *ν_max_* 1493, 1498, 1598, 1621, 1650, 1680, 3252 cm^-1^; ^1^H NMR (500 MHz, CDCl_3_ + CD_3_OD): δ (ppm) 8.82 (d, *J* = 10 Hz, 1H), 7.28 (d, *J* = 10 Hz, 1H), 7.14 (m, 2H), 6.45 (s, 1H), 6.14 (s, 1H), 2.12 (s, 3H); ^13^C NMR (125 MHz, CDCl_3_+ CD_3_OD): δ (ppm) 181.80, 163.82, 160.84, 157.71, 156.08, 150.01, 129.93, 128.10, 123.68, 118.70, 118.24, 110.66, 109.77, 106.37, 94.43, 89.91, 8.27 HR-ESIMS: *m/z* 313.0708 calculated for C_17_H_12_O6+H^+^ (313.0707).

**^1^**H NMR scan of boeravinone B:

| 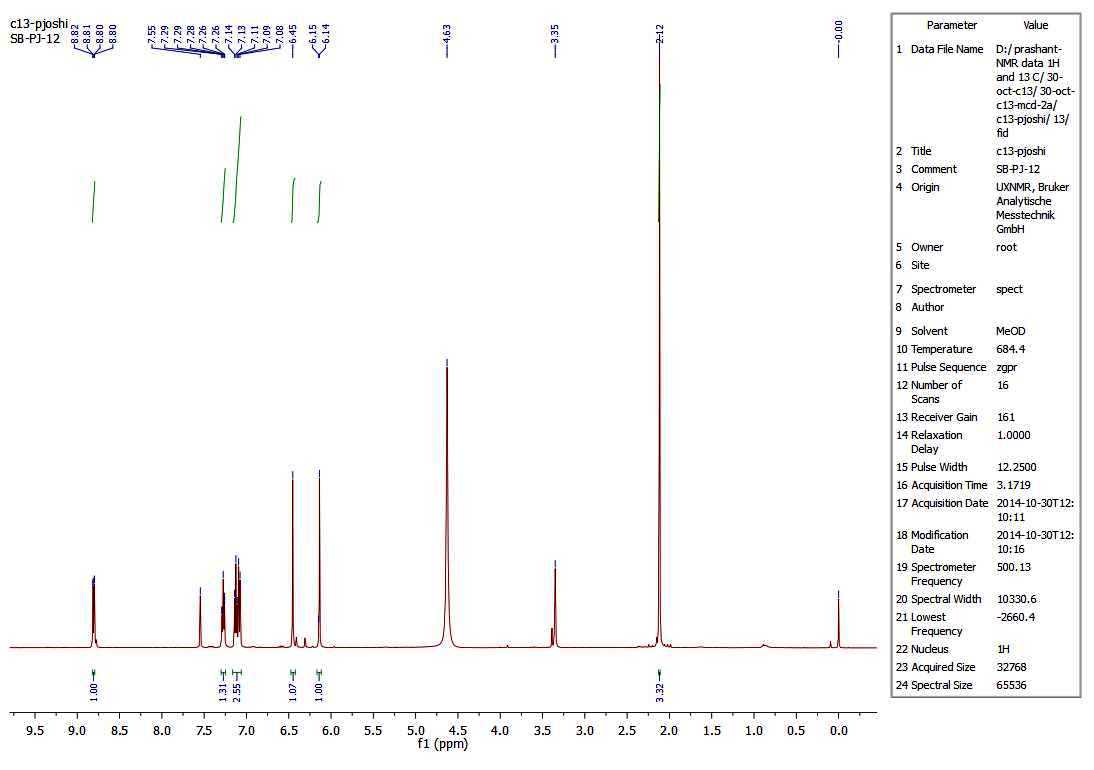 |
| --- |

**^13^**C NMR scan of boeravinone B:

|  |
| --- |
| 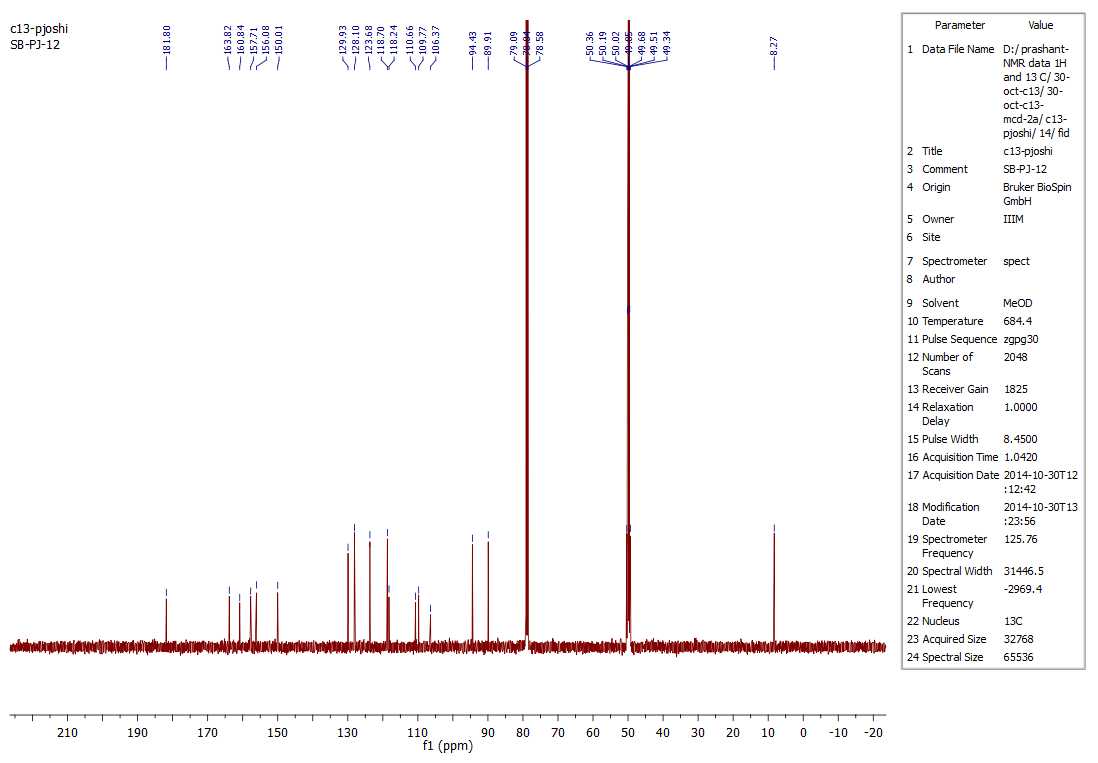 |

DEPT135 NMR scan of boeravinone B:

|  |
| --- |
| 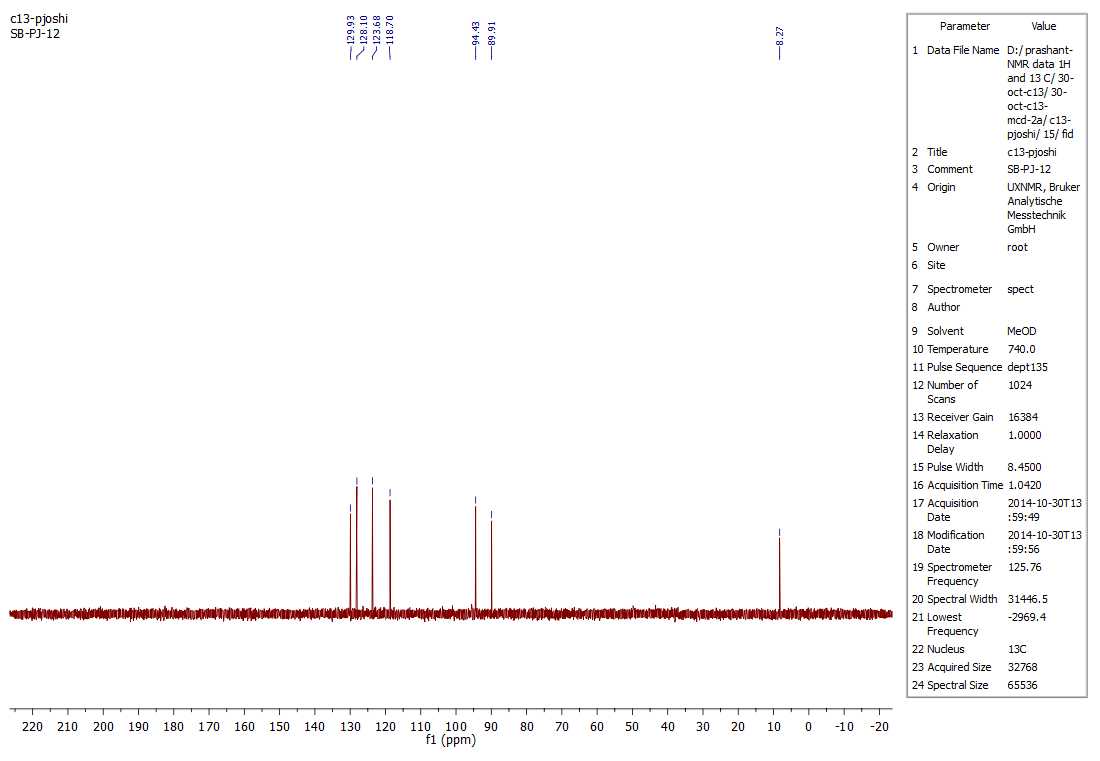 |

**HR-ESIMS scan of boeravinone B:**

**HPLC spectra of boeravinone B:**

**Figure S1:** Biofilm inhibition by ciprofloxacin alone and in combination using SA-1199B (a) and SA-1199 (b). After incubation resazurin was added to wells to enumerate the viable cells in biofilm. Assay was performed according to previously described method (2).

**(a)**


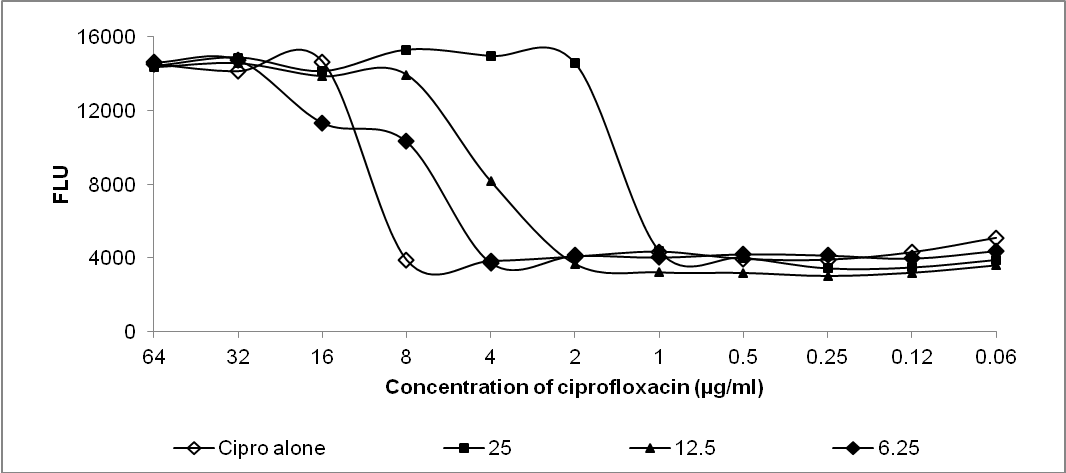


**(b)**
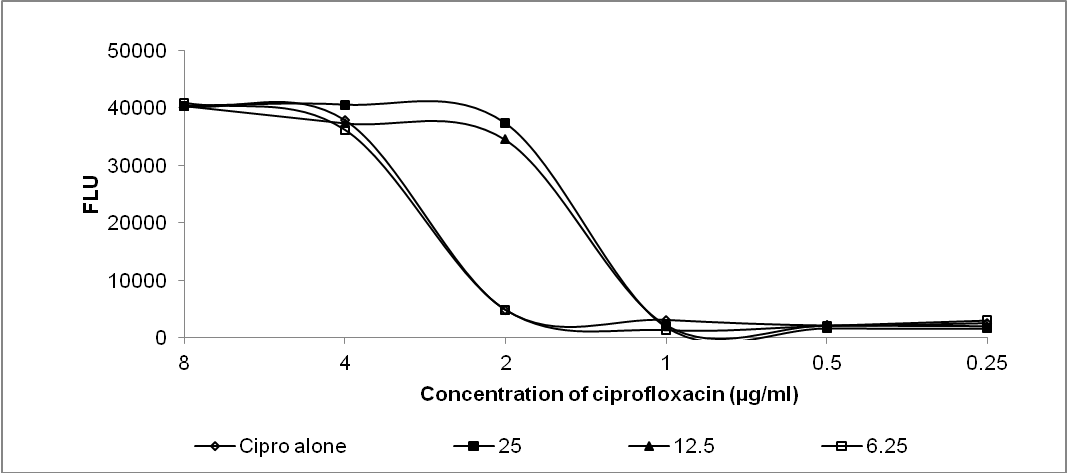


**References:**

1. Kadota S, Tezuka NY, Kikuchi T. 1989. [Constituents of the Roots of *Boerhaavia diffusa* L.I. examination of sterols and structures of new rotenoids, boeravinones A and B](https://www.jstage.jst.go.jp/A_PRedirectJournalInit?sryCd=cpb1958&noVol=37&noIssue=12&kijiCd=37_12_3214&screenID=AF06S010). Chem Pharma Bull 37:3214–3220.
2. Bauer J, Siala W, Tulkens PM, Van Bambeke F. 2013. A combined pharmacodynamic quantitative and qualitative model reveals the potent activity of daptomycin and delafloxacin against *Staphylococcus aureus* biofilms. Antimicrob. Agents Chemother 57:2726–2637.
